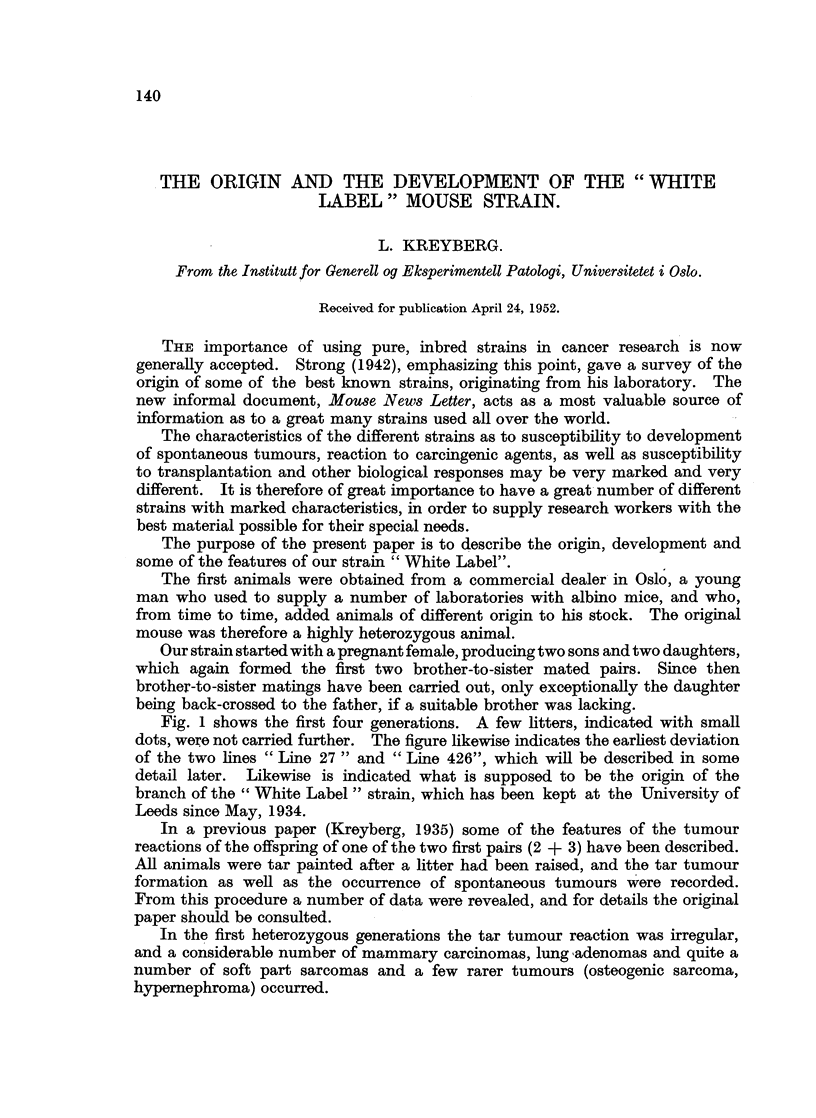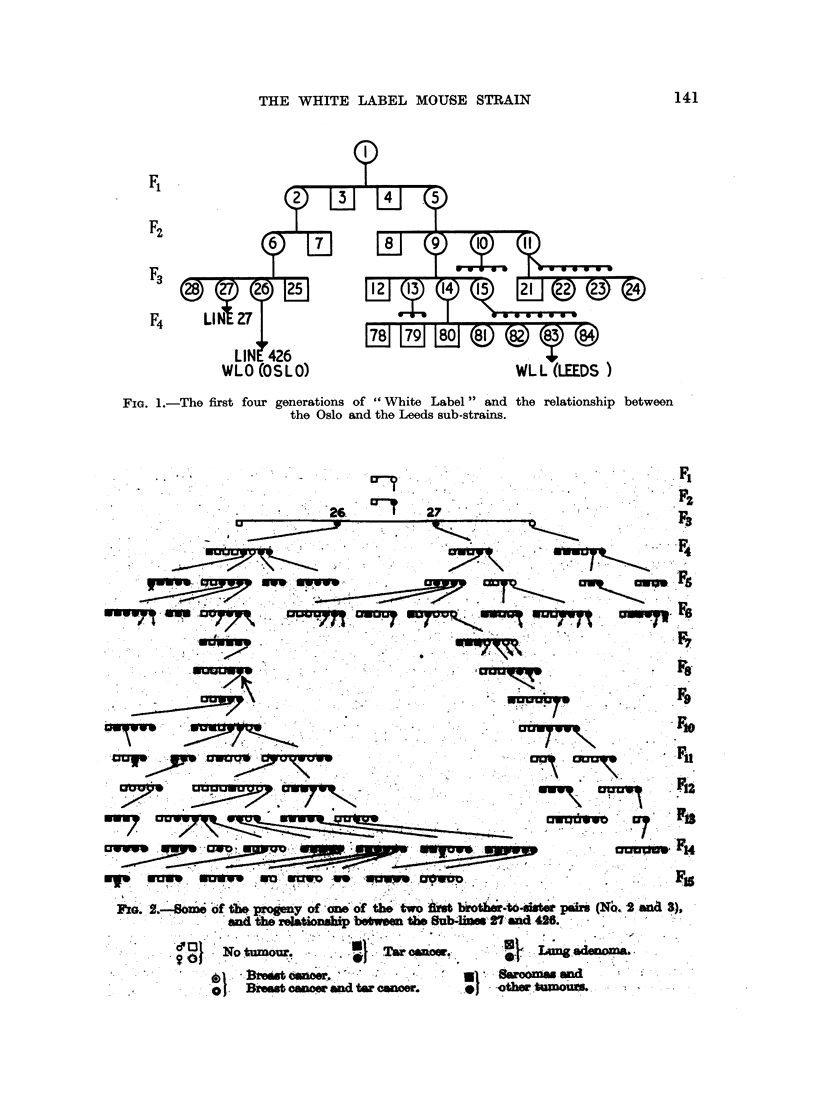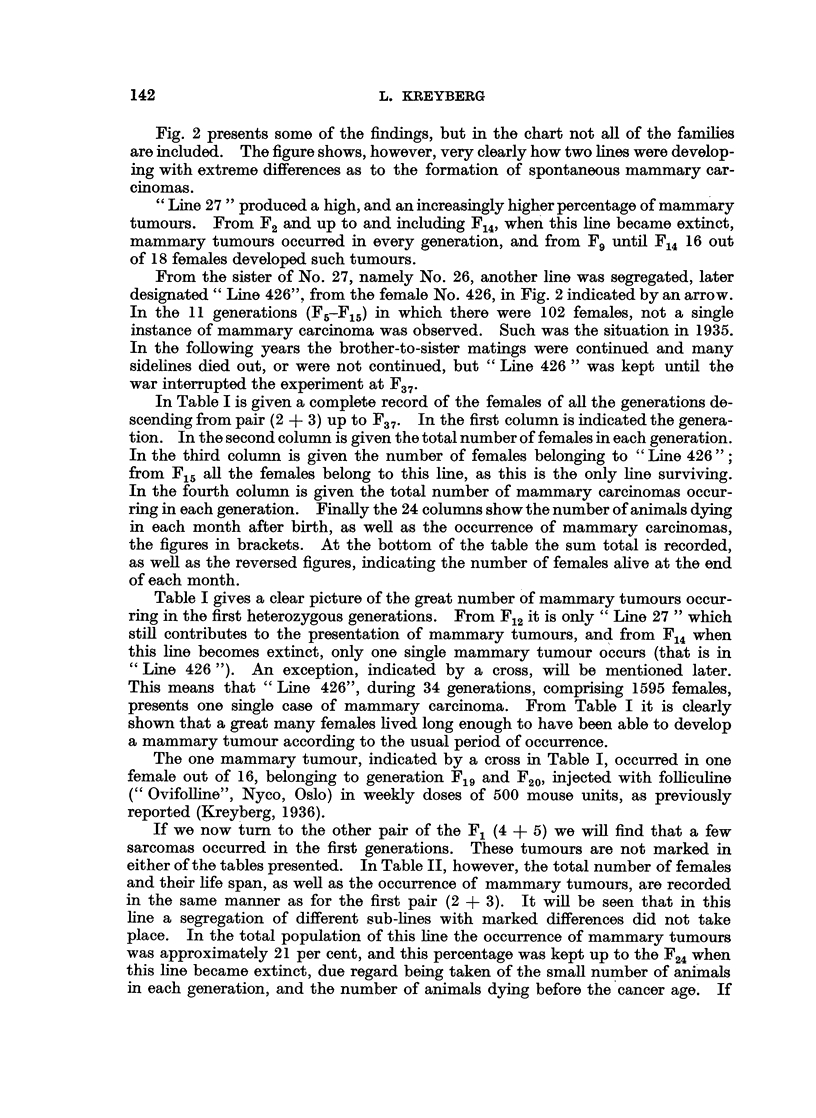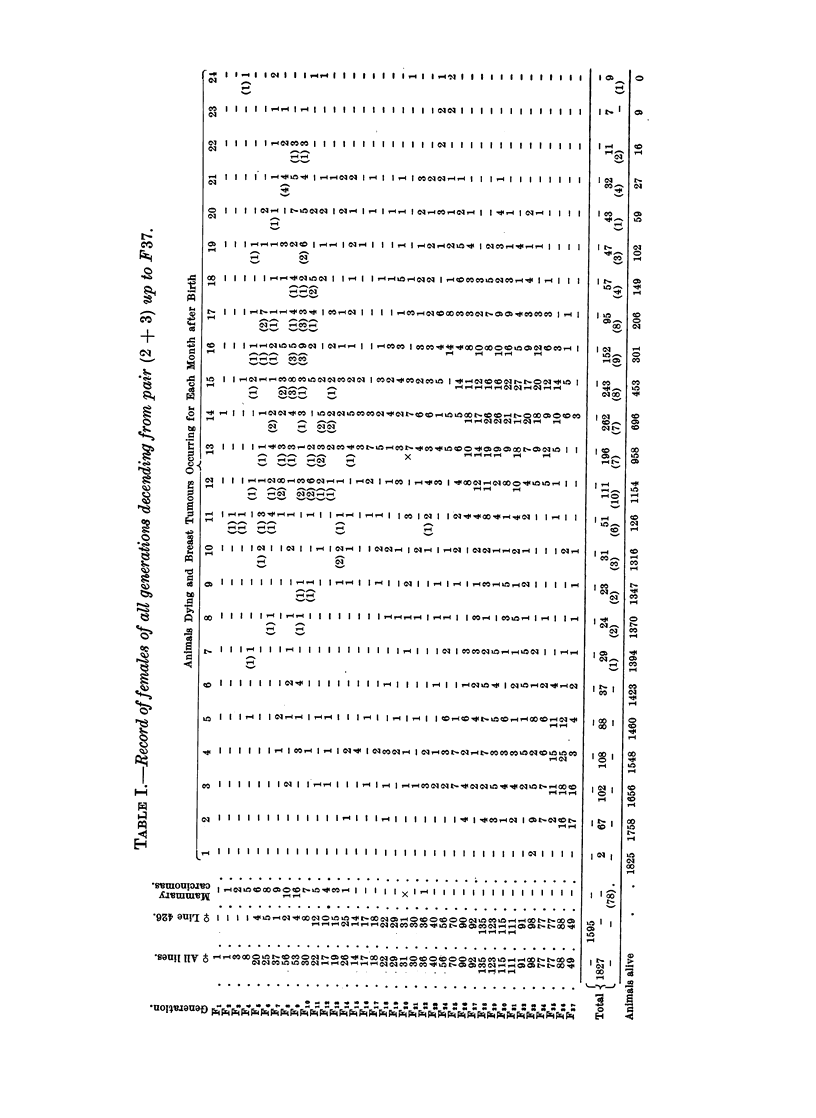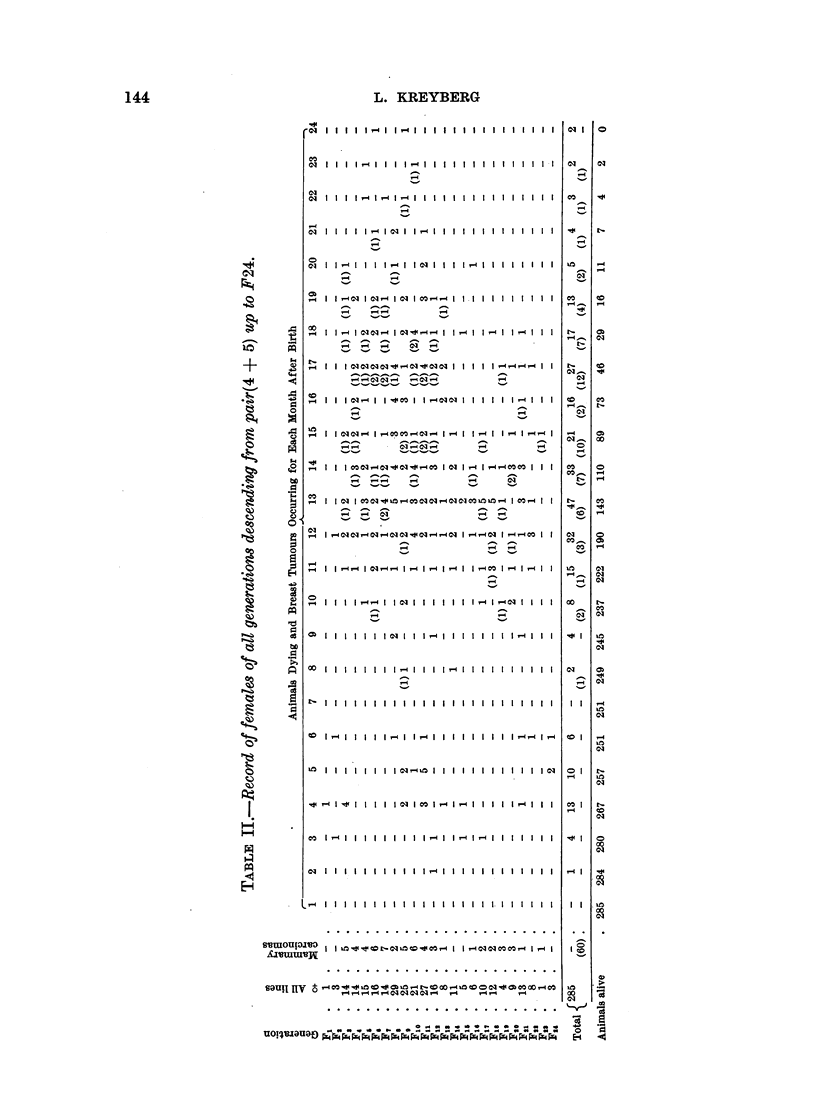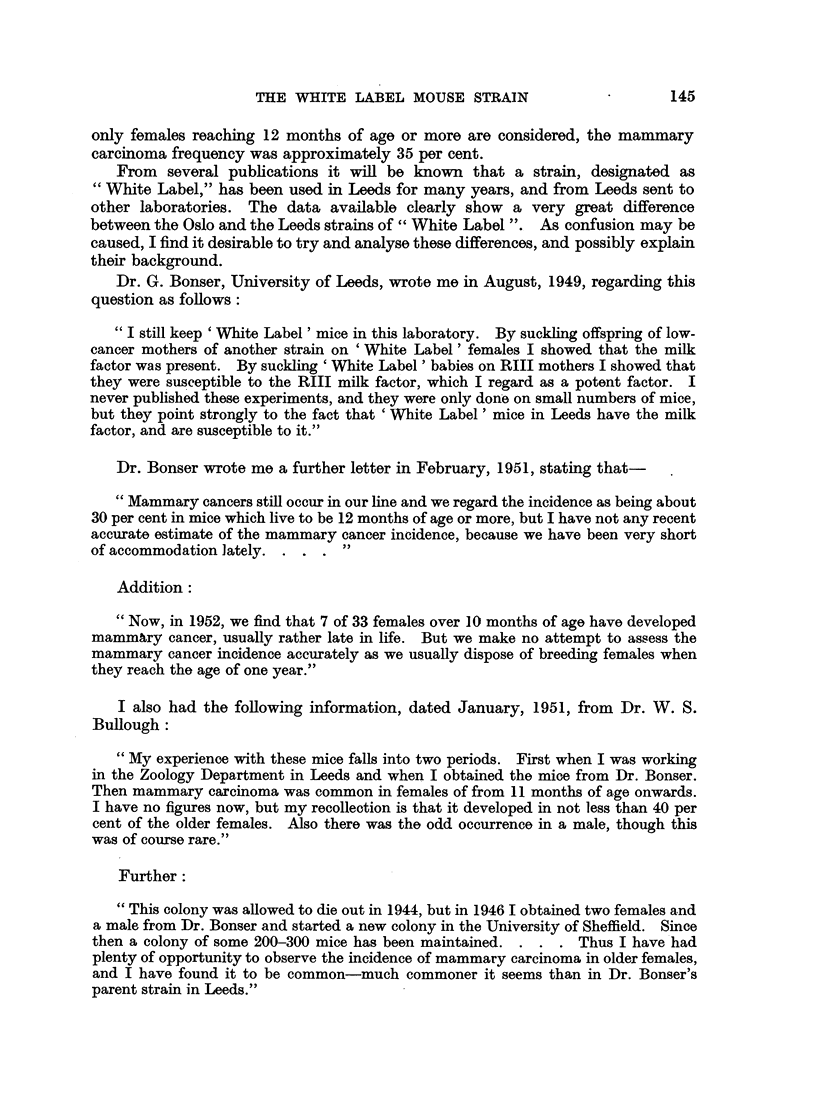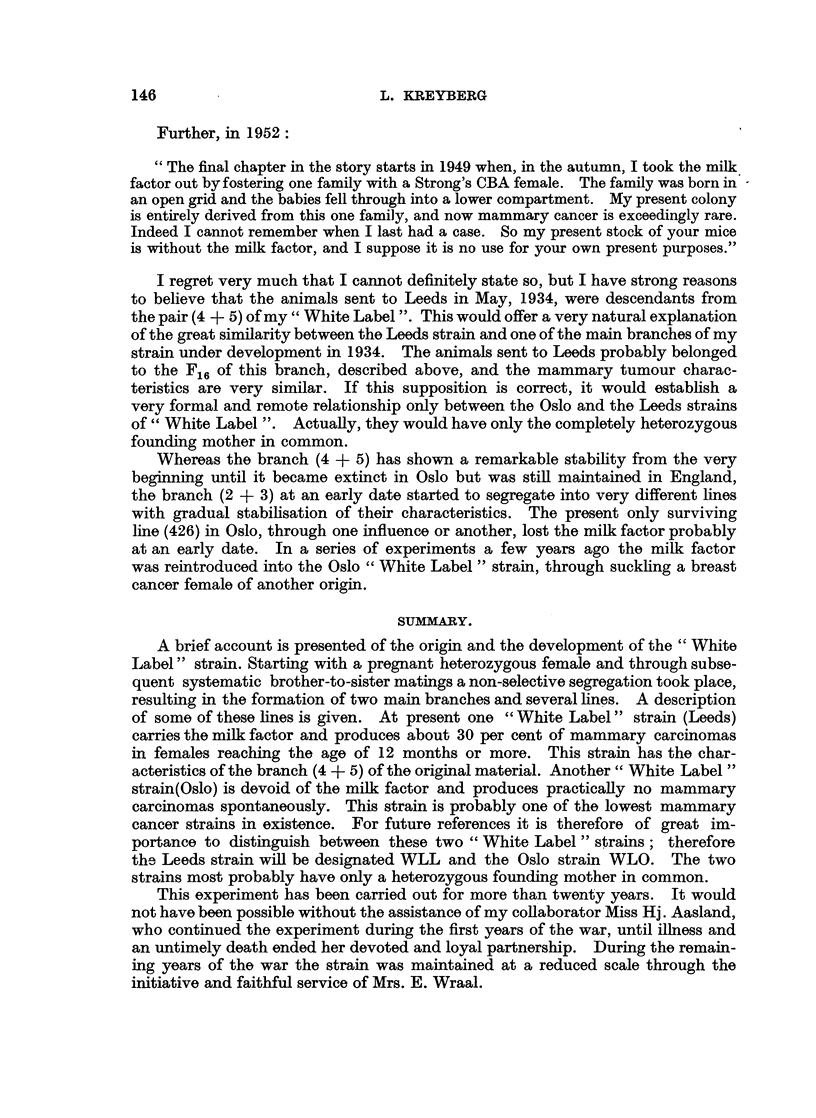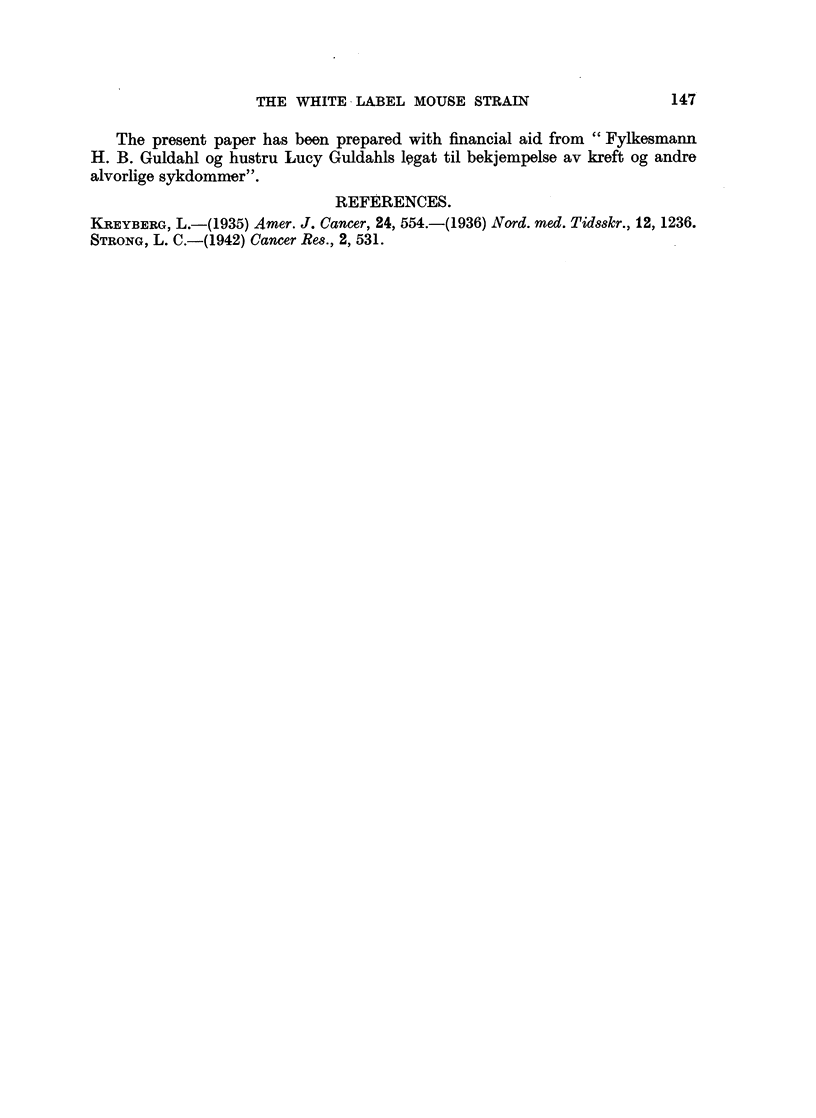# The Origin and the Development of the “White Label” Mouse Strain

**DOI:** 10.1038/bjc.1952.16

**Published:** 1952-06

**Authors:** L. Kreyberg


### THE ORIGIN AND THE DEVELOPMENT OF THE "WHITE LABEL" MOUSE STRAIN.

L. KREYBERG.

*From the Institutt for Generell og Eksperimentell Patologi, Universitetet i Oslo.*

Received for publication April 24, 1952.

THE importance of using pure, inbred strains in cancer research is now generally accepted. Strong (1942), emphasizing this point, gave a survey of the origin of some of the best known strains, originating from his laboratory. The new informal document, *Mouse News Letter*, acts as a most valuable source of information as to a great many strains used all over the world.

The characteristics of the different strains as to susceptibility to development of spontaneous tumours, reaction to carcingenic agents, as well as susceptibility to transplantation and other biological responses may be very marked and very different. It is therefore of great importance to have a great number of different strains with marked characteristics, in order to supply research workers with the best material possible for their special needs.

The purpose of the present paper is to describe the origin, development and some of the features of our strain " White Label".

The first animals were obtained from a commercial dealer in Oslo, a young man who used to supply a number of laboratories with albino mice, and who, from time to time, added animals of different origin to his stock. The original mouse was therefore a highly heterozygous animal.

Our strain started with a pregnant female, producing two sons and two daughters, which again formed the first two brother-to-sister mated pairs. Since then brother-to-sister matings have been carried out, only exceptionally the daughter being back-crossed to the father, if a suitable brother was lacking.

Fig. 1 shows the first four generations. A few litters, indicated with small dots, were not carried further. The figure likewise indicates the earliest deviation of the two lines " Line 27 " and " Line 426", which will be described in some detail later. Likewise is indicated what is supposed to be the origin of the branch of the " White Label " strain, which has been kept at the University of Leeds since May, 1934.

In a previous paper (Kreyberg, 1935) some of the features of the tumour reactions of the offspring of one of the two first pairs (2 + 3) have been described. All animals were tar painted after a litter had been raised, and the tar tumour formation as well as the occurrence of spontaneous tumours were recorded. From this procedure a number of data were revealed, and for details the original paper should be consulted.

In the first heterozygous generations the tar tumour reaction was irregular, and a considerable number of mammary carcinomas, lung adenomas and quite a number of soft part sarcomas and a few rarer tumours (osteogenic sarcoma, hypernephroma) occurred.

FIG. 1.—The first four generations of "White Label" and the relationship between the Oslo and the Leeds sub-strains.

FIG. 2.—Some of the progeny of one of the two first brother-to-sister pairs (No. 2 and 3), and the relationship between the Sub-lines 27 and 426.

♂ □ ♀ ○ } No tumour. ■ ● } Tar cancer. ⊠ ⊗ } Lung adenoma.

⊙ ○ } Breast cancer. Breast cancer and tar cancer. ▥ ◍ } Sarcomas and other tumours.

Fig. 2 presents some of the findings, but in the chart not all of the families are included. The figure shows, however, very clearly how two lines were developing with extreme differences as to the formation of spontaneous mammary carcinomas.

" Line 27 " produced a high, and an increasingly higher percentage of mammary tumours. From $F_2$ and up to and including $F_{14}$, when this line became extinct, mammary tumours occurred in every generation, and from $F_9$ until $F_{14}$ 16 out of 18 females developed such tumours.

From the sister of No. 27, namely No. 26, another line was segregated, later designated " Line 426", from the female No. 426, in Fig. 2 indicated by an arrow. In the 11 generations ($F_5$–$F_{15}$) in which there were 102 females, not a single instance of mammary carcinoma was observed. Such was the situation in 1935. In the following years the brother-to-sister matings were continued and many sidelines died out, or were not continued, but " Line 426 " was kept until the war interrupted the experiment at $F_{37}$.

In Table I is given a complete record of the females of all the generations descending from pair (2 + 3) up to $F_{37}$. In the first column is indicated the generation. In the second column is given the total number of females in each generation. In the third column is given the number of females belonging to "Line 426"; from $F_{15}$ all the females belong to this line, as this is the only line surviving. In the fourth column is given the total number of mammary carcinomas occurring in each generation. Finally the 24 columns show the number of animals dying in each month after birth, as well as the occurrence of mammary carcinomas, the figures in brackets. At the bottom of the table the sum total is recorded, as well as the reversed figures, indicating the number of females alive at the end of each month.

Table I gives a clear picture of the great number of mammary tumours occurring in the first heterozygous generations. From $F_{12}$ it is only " Line 27 " which still contributes to the presentation of mammary tumours, and from $F_{14}$ when this line becomes extinct, only one single mammary tumour occurs (that is in " Line 426 "). An exception, indicated by a cross, will be mentioned later. This means that " Line 426", during 34 generations, comprising 1595 females, presents one single case of mammary carcinoma. From Table I it is clearly shown that a great many females lived long enough to have been able to develop a mammary tumour according to the usual period of occurrence.

The one mammary tumour, indicated by a cross in Table I, occurred in one female out of 16, belonging to generation $F_{19}$ and $F_{20}$, injected with folliculine (" Ovifolline", Nyco, Oslo) in weekly doses of 500 mouse units, as previously reported (Kreyberg, 1936).

If we now turn to the other pair of the $F_1$ (4 + 5) we will find that a few sarcomas occurred in the first generations. These tumours are not marked in either of the tables presented. In Table II, however, the total number of females and their life span, as well as the occurrence of mammary tumours, are recorded in the same manner as for the first pair (2 + 3). It will be seen that in this line a segregation of different sub-lines with marked differences did not take place. In the total population of this line the occurrence of mammary tumours was approximately 21 per cent, and this percentage was kept up to the $F_{24}$ when this line became extinct, due regard being taken of the small number of animals in each generation, and the number of animals dying before the cancer age. If

### Table I.—*Record of females of all generations decending from pair (2 + 3) up to F37.*

| Generation. | ♀ All lines. | ♀ Line 426. | Mammary carcinomas. | Animals Dying and Breast Tumours Occurring for Each Month after Birth | | | | | | | | | | | | | | | | | | | | | | | |
|---|---|---|---|---|---|---|---|---|---|---|---|---|---|---|---|---|---|---|---|---|---|---|---|---|---|---|---|
| | | | | 1 | 2 | 3 | 4 | 5 | 6 | 7 | 8 | 9 | 10 | 11 | 12 | 13 | 14 | 15 | 16 | 17 | 18 | 19 | 20 | 21 | 22 | 23 | 24 |
| $F_1$ | 1 | – | – | – | – | – | – | – | – | – | – | – | – | – | – | – | 1 | – | – | – | – | – | – | – | – | – | – |
| $F_2$ | 1 | – | 1 | – | – | – | – | – | – | – | – | – | – | (1) 1 | – | – | – | – | – | – | – | – | – | – | – | – | – |
| $F_3$ | 3 | – | 2 | – | – | – | – | – | – | – | – | – | – | (1) 1 | – | – | – | 1 | – | – | – | – | – | – | – | – | (1) 1 |
| $F_4$ | 8 | – | 5 | – | – | – | – | 1 | – | (1) 1 | – | – | – | – | (1) 1 | – | – | (1) 2 | (1) 1 | 1 | – | (1) 1 | – | – | – | – | – |
| $F_5$ | 20 | 4 | 6 | – | – | – | – | – | – | – | – | – | (1) 2 | (1) 3 | 1 | (1) 1 | 1 | 1 | (1) 1 | (2) 7 | – | 1 | 2 | – | – | – | – |
| $F_6$ | 25 | 5 | 8 | – | – | – | – | – | – | – | (1) 1 | – | – | (1) 4 | (1) 2 | 4 | (2) 2 | 1 | (1) 2 | (1) 1 | 1 | 1 | (1) 1 | 1 | 1 | 1 | 2 |
| $F_7$ | 37 | 1 | 9 | – | – | – | 1 | 2 | – | – | – | – | – | 1 | (2) 8 | (1) 3 | 2 | (2) 3 | 5 | 1 | 1 | 3 | – | (4) 4 | 2 | 1 | – |
| $F_8$ | 56 | 2 | 10 | – | – | 2 | – | 1 | 2 | 1 | 1 | – | 2 | 1 | 1 | (1) 3 | 4 | (3) 8 | (3) 5 | (1) 4 | (1) 4 | 2 | 7 | 5 | (1) 3 | – | – |
| $F_9$ | 53 | 4 | 16 | – | – | – | 3 | 1 | 4 | – | (1) 1 | (1) 1 | – | – | (2) 3 | 1 | (1) 3 | (1) 3 | (3) 9 | (3) 3 | (1) 2 | (2) 6 | 5 | 4 | (1) 3 | 1 | – |
| $F_{10}$ | 30 | 8 | 7 | – | – | – | 1 | – | – | – | – | (1) 1 | – | 1 | (2) 6 | (1) 2 | – | 5 | 2 | (1) 4 | (2) 5 | – | 2 | – | – | – | 1 |
| $F_{11}$ | 22 | 12 | 5 | – | – | 1 | – | 1 | – | – | – | – | 1 | – | (1) 2 | (2) 3 | (2) 5 | 2 | – | – | 2 | 1 | 2 | 1 | – | – | 1 |
| $F_{12}$ | 17 | 10 | 4 | – | – | 1 | 1 | 1 | – | – | – | – | – | – | (1) 1 | 2 | (2) 2 | (1) 2 | 2 | 3 | – | 1 | – | 1 | – | – | – |
| $F_{13}$ | 19 | 15 | 3 | – | – | – | – | – | – | – | – | 1 | (2) 2 | (1) 1 | 1 | 3 | 2 | 3 | 1 | 1 | – | – | 2 | 2 | – | – | – |
| $F_{14}$ | 26 | 25 | 1 | – | 1 | – | 2 | – | – | – | – | 1 | 1 | 1 | – | (1) 4 | 5 | 2 | 1 | 2 | 1 | 2 | 1 | 2 | – | – | – |
| $F_{15}$ | 14 | 14 | – | – | – | – | 4 | – | – | – | – | – | – | – | 1 | 3 | 3 | 2 | – | – | – | 1 | – | – | – | – | – |
| $F_{16}$ | 17 | 17 | – | – | – | 1 | – | 1 | – | – | – | – | – | 1 | 2 | 7 | 3 | – | – | – | – | – | 1 | 1 | – | – | – |
| $F_{17}$ | 18 | 18 | – | – | – | – | 2 | – | – | – | – | 1 | 2 | 1 | – | 5 | 2 | 3 | 1 | – | 1 | – | – | – | – | – | – |
| $F_{18}$ | 22 | 22 | – | – | 1 | 1 | 3 | – | 1 | – | 1 | – | 2 | – | 1 | 1 | 4 | 2 | 3 | – | 1 | – | 1 | – | – | – | – |
| $F_{19}$ | 29 | 29 | – | – | – | – | 2 | 1 | – | – | 1 | – | 1 | – | 3 | 3 | 2 | 4 | 3 | 1 | 5 | 1 | 1 | 1 | – | – | – |
| $F_{20}$ | 31 | 31 | × | – | – | 1 | 1 | – | – | 1 | 1 | 2 | – | 3 | – | ×7 | 7 | 3 | – | 3 | 1 | – | – | – | – | – | 1 |
| $F_{21}$ | 30 | 30 | – | – | – | 1 | – | 1 | – | – | 1 | – | 2 | – | 1 | 4 | 6 | 2 | 3 | 1 | 2 | 1 | 2 | 3 | – | – | – |
| $F_{22}$ | 36 | 36 | 1 | – | – | 3 | 2 | – | – | – | – | – | 1 | (1) 2 | 4 | 3 | 6 | 3 | 3 | 2 | 2 | 2 | 1 | 2 | – | – | – |
| $F_{23}$ | 40 | 40 | – | – | – | 2 | 1 | – | 1 | – | 1 | 1 | – | – | 3 | 4 | 1 | 5 | 4 | 6 | – | 1 | 3 | 2 | 2 | 2 | 1 |
| $F_{24}$ | 56 | 56 | – | – | – | 2 | 3 | 6 | – | 2 | 1 | – | 1 | – | – | 5 | 5 | – | 14 | 8 | 1 | 2 | 1 | 1 | – | 2 | 2 |
| $F_{25}$ | 70 | 70 | – | – | – | 7 | 7 | 1 | – | – | – | 1 | 2 | 2 | 4 | 6 | 5 | 14 | 4 | 3 | 6 | 5 | 2 | 1 | – | – | – |
| $F_{26}$ | 90 | 90 | – | – | 4 | 4 | 2 | 6 | 1 | 3 | – | – | – | 4 | 8 | 10 | 18 | 11 | 8 | 3 | 3 | 4 | 1 | – | – | – | – |
| $F_{27}$ | 92 | 92 | – | – | – | 2 | 1 | 4 | 2 | 3 | 3 | 1 | 2 | 4 | 12 | 14 | 17 | 12 | 10 | 2 | 3 | – | – | – | – | – | – |
| $F_{28}$ | 135 | 135 | – | – | 4 | 2 | 7 | 7 | 5 | 2 | 1 | 3 | 2 | 8 | 11 | 19 | 26 | 16 | 8 | 7 | 5 | 2 | – | – | – | – | – |
| $F_{29}$ | 123 | 123 | – | – | 3 | 5 | 3 | 5 | 4 | 5 | – | 1 | 1 | 4 | 2 | 19 | 26 | 16 | 10 | 9 | 2 | 3 | 4 | 1 | – | – | – |
| $F_{30}$ | 115 | 115 | – | – | 1 | 4 | 3 | 6 | – | 1 | 3 | 5 | 1 | 1 | 8 | 9 | 21 | 22 | 16 | 9 | 3 | 1 | 1 | – | – | – | – |
| $F_{31}$ | 111 | 111 | – | – | 2 | 4 | 3 | 1 | 2 | 1 | 5 | 1 | 2 | 4 | 10 | 18 | 17 | 27 | 5 | 4 | 1 | 4 | – | – | – | – | – |
| $F_{32}$ | 91 | 91 | – | – | – | 2 | 5 | 1 | 5 | 5 | 1 | 2 | 1 | 2 | 4 | 7 | 20 | 17 | 9 | 3 | 4 | 1 | 2 | – | – | – | – |
| $F_{33}$ | 98 | 98 | – | 2 | 9 | 5 | 2 | 8 | 1 | 2 | – | – | – | – | 5 | 9 | 18 | 20 | 12 | 3 | – | 1 | 1 | – | – | – | – |
| $F_{34}$ | 77 | 77 | – | – | 7 | 7 | 6 | 6 | 2 | – | 1 | – | – | – | 5 | 12 | 9 | 12 | 6 | 3 | 1 | – | – | – | – | – | – |
| $F_{35}$ | 77 | 77 | – | – | 2 | 11 | 15 | 11 | 4 | – | – | – | – | 1 | 1 | 5 | 10 | 14 | 3 | – | – | – | – | – | – | – | – |
| $F_{36}$ | 88 | 88 | – | – | 16 | 18 | 25 | 12 | 1 | 1 | – | – | 2 | – | – | – | 6 | 5 | 1 | 1 | – | – | – | – | – | – | – |
| $F_{37}$ | 49 | 49 | – | – | 17 | 16 | 3 | 4 | 2 | 1 | 1 | 1 | 1 | – | – | – | 3 | – | – | – | – | – | – | – | – | – | – |
| Total | – | 1595 | – | – | – | – | – | – | – | – | – | – | – | – | – | – | – | – | – | – | – | – | – | – | – | – | – |
| | 1827 | – | – | 2 | 67 | 102 | 108 | 88 | 37 | 29 | 24 | 23 | 31 | 51 | 111 | 196 | 262 | 243 | 152 | 95 | 57 | 47 | 43 | 32 | 11 | 7 | 9 |
| | – | – | (78). | – | – | – | – | – | – | (1) | (2) | (2) | (3) | (6) | (10) | (7) | (7) | (8) | (9) | (8) | (4) | (3) | (1) | (4) | (2) | – | (1) |
| Animals alive | | | | 1825 | 1758 | 1656 | 1548 | 1460 | 1423 | 1394 | 1370 | 1347 | 1316 | 126 | 1154 | 958 | 696 | 453 | 301 | 206 | 149 | 102 | 59 | 27 | 16 | 9 | 0 |

TABLE II.—*Record of females of all generations descending from pair(4 + 5) up to F24.*

Animals Dying and Breast Tumours Occurring for Each Month After Birth (months 1–12)

| Generation | ♀ All lines | Mammary carcinomas | 1 | 2 | 3 | 4 | 5 | 6 | 7 | 8 | 9 | 10 | 11 | 12 |
|---|---|---|---|---|---|---|---|---|---|---|---|---|---|---|
| $F_1$ | 1 | – | – | – | – | 1 | – | – | – | – | – | – | – | – |
| $F_2$ | 3 | – | – | – | 1 | – | – | 1 | – | – | – | – | – | 1 |
| $F_3$ | 14 | 5 | – | – | – | 4 | – | – | – | – | – | – | 1 | 2 |
| $F_4$ | 14 | 4 | – | – | – | – | – | – | – | – | – | – | 1 | 2 |
| $F_5$ | 15 | 4 | – | – | – | – | – | – | – | – | – | 1 | – | 1 |
| $F_6$ | 16 | 6 | – | – | – | – | – | – | – | – | – | (1) 1 | 2 | 2 |
| $F_7$ | 14 | 7 | – | – | – | – | – | – | – | – | – | – | 1 | 1 |
| $F_8$ | 29 | 2 | – | – | – | – | – | 1 | – | – | 2 | – | 1 | 2 |
| $F_9$ | 25 | 5 | – | – | – | 2 | 2 | – | – | (1) 1 | – | 2 | – | (1) 2 |
| $F_{10}$ | 21 | 6 | – | – | – | – | 1 | – | – | – | – | – | 1 | 4 |
| $F_{11}$ | 27 | 4 | – | – | – | 3 | 5 | 1 | – | – | – | – | – | 2 |
| $F_{12}$ | 16 | 3 | – | 1 | 1 | – | – | – | – | – | 1 | – | 1 | 1 |
| $F_{13}$ | 8 | 1 | – | – | – | 1 | – | – | – | – | – | – | – | 1 |
| $F_{14}$ | 11 | – | – | – | – | – | – | – | – | 1 | – | – | 1 | 2 |
| $F_{15}$ | 5 | – | – | – | 1 | 1 | – | – | – | – | – | – | – | – |
| $F_{16}$ | 6 | 1 | – | – | – | – | – | – | – | – | – | – | – | 1 |
| $F_{17}$ | 10 | 2 | – | – | 1 | – | – | – | – | – | – | 1 | 1 | 1 |
| $F_{18}$ | 12 | 2 | – | – | – | – | – | – | – | – | – | – | (1) 3 | (1) 2 |
| $F_{19}$ | 4 | 3 | – | – | – | – | – | – | – | – | – | (1) 1 | – | – |
| $F_{20}$ | 9 | 3 | – | – | – | – | – | – | – | – | – | 2 | 1 | (1) 1 |
| $F_{21}$ | 13 | 1 | – | – | – | 1 | – | 1 | – | – | 1 | – | – | 1 |
| $F_{22}$ | 8 | – | – | – | – | – | – | 1 | – | – | – | – | 1 | 3 |
| $F_{23}$ | 1 | 1 | – | – | – | – | – | – | – | – | – | – | – | – |
| $F_{24}$ | 3 | – | – | – | – | – | 2 | 1 | – | – | – | – | – | – |
| Total | 285 | – | – | 1 | 4 | 13 | 10 | 6 | – | 2 | 4 | 8 | 15 | 32 |
| | | (60) | – | – | – | – | – | – | – | (1) | – | (2) | (1) | (3) |
| Animals alive | | | 285 | 284 | 280 | 267 | 257 | 251 | 251 | 249 | 245 | 237 | 222 | 190 |

Animals Dying and Breast Tumours Occurring for Each Month After Birth (months 13–24)

| Generation | ♀ All lines | Mammary carcinomas | 13 | 14 | 15 | 16 | 17 | 18 | 19 | 20 | 21 | 22 | 23 | 24 |
|---|---|---|---|---|---|---|---|---|---|---|---|---|---|---|
| $F_1$ | 1 | – | – | – | – | – | – | – | – | – | – | – | – | – |
| $F_2$ | 3 | – | – | – | – | – | – | – | – | – | – | – | – | – |
| $F_3$ | 14 | 5 | (1) 2 | – | (1) 2 | – | – | (1) 1 | (1) 1 | (1) 1 | – | – | – | – |
| $F_4$ | 14 | 4 | – | (1) 3 | (1) 2 | (1) 2 | (1) 2 | – | 2 | – | – | – | – | – |
| $F_5$ | 15 | 4 | (1) 3 | 2 | 1 | 1 | (1) 2 | (1) 2 | – | – | – | 1 | 1 | – |
| $F_6$ | 16 | 6 | 2 | (1) 1 | – | – | (2) 2 | 2 | (1) 2 | – | (1) 1 | – | – | 1 |
| $F_7$ | 14 | 7 | (2) 4 | (1) 2 | 1 | – | (2) 2 | (1) 1 | (1) 1 | – | – | 1 | – | – |
| $F_8$ | 29 | 2 | 5 | 4 | 3 | 4 | (1) 4 | – | – | (1) 1 | 2 | – | – | – |
| $F_9$ | 25 | 5 | 1 | 2 | (2) 3 | 3 | 1 | 2 | 2 | – | – | (1) 1 | – | 1 |
| $F_{10}$ | 21 | 6 | 3 | (1) 4 | (1) 1 | – | (1) 2 | (2) 4 | – | – | – | – | (1) 1 | – |
| $F_{11}$ | 27 | 4 | 2 | 1 | (2) 2 | – | (2) 4 | 1 | 3 | 2 | 1 | – | – | – |
| $F_{12}$ | 16 | 3 | 2 | 3 | (1) 1 | 1 | (1) 2 | (1) 1 | 1 | – | – | – | – | – |
| $F_{13}$ | 8 | 1 | 1 | – | – | 2 | 2 | – | (1) 1 | – | – | – | – | – |
| $F_{14}$ | 11 | – | 2 | 2 | 1 | 2 | – | – | – | – | – | – | – | – |
| $F_{15}$ | 5 | – | 2 | – | – | – | – | 1 | – | – | – | – | – | – |
| $F_{16}$ | 6 | 1 | 3 | (1) 1 | – | – | – | – | – | 1 | – | – | – | – |
| $F_{17}$ | 10 | 2 | (1) 5 | – | (1) 1 | – | – | – | – | – | – | – | – | – |
| $F_{18}$ | 12 | 2 | 5 | 1 | – | – | – | 1 | – | – | – | – | – | – |
| $F_{19}$ | 4 | 3 | (1) 1 | 1 | – | – | (1) 1 | – | – | – | – | – | – | – |
| $F_{20}$ | 9 | 3 | – | (2) 3 | 1 | – | 1 | – | – | – | – | – | – | – |
| $F_{21}$ | 13 | 1 | 3 | 3 | – | (1) 1 | 1 | 1 | – | – | – | – | – | – |
| $F_{22}$ | 8 | – | 1 | – | 1 | – | 1 | – | – | – | – | – | – | – |
| $F_{23}$ | 1 | 1 | – | – | (1) 1 | – | – | – | – | – | – | – | – | – |
| $F_{24}$ | 3 | – | – | – | – | – | – | – | – | – | – | – | – | – |
| Total | 285 | – | 47 | 33 | 21 | 16 | 27 | 17 | 13 | 5 | 4 | 3 | 2 | 2 |
| | | (60) | (6) | (7) | (10) | (2) | (12) | (7) | (4) | (2) | (1) | (1) | (1) | – |
| Animals alive | | | 143 | 110 | 89 | 73 | 46 | 29 | 16 | 11 | 7 | 4 | 2 | 0 |

only females reaching 12 months of age or more are considered, the mammary carcinoma frequency was approximately 35 per cent.

From several publications it will be known that a strain, designated as "White Label," has been used in Leeds for many years, and from Leeds sent to other laboratories. The data available clearly show a very great difference between the Oslo and the Leeds strains of "White Label". As confusion may be caused, I find it desirable to try and analyse these differences, and possibly explain their background.

Dr. G. Bonser, University of Leeds, wrote me in August, 1949, regarding this question as follows :

"I still keep 'White Label' mice in this laboratory. By suckling offspring of low-cancer mothers of another strain on 'White Label' females I showed that the milk factor was present. By suckling 'White Label' babies on RIII mothers I showed that they were susceptible to the RIII milk factor, which I regard as a potent factor. I never published these experiments, and they were only done on small numbers of mice, but they point strongly to the fact that 'White Label' mice in Leeds have the milk factor, and are susceptible to it."

Dr. Bonser wrote me a further letter in February, 1951, stating that—

"Mammary cancers still occur in our line and we regard the incidence as being about 30 per cent in mice which live to be 12 months of age or more, but I have not any recent accurate estimate of the mammary cancer incidence, because we have been very short of accommodation lately. . . ."

Addition :

"Now, in 1952, we find that 7 of 33 females over 10 months of age have developed mammary cancer, usually rather late in life. But we make no attempt to assess the mammary cancer incidence accurately as we usually dispose of breeding females when they reach the age of one year."

I also had the following information, dated January, 1951, from Dr. W. S. Bullough :

"My experience with these mice falls into two periods. First when I was working in the Zoology Department in Leeds and when I obtained the mice from Dr. Bonser. Then mammary carcinoma was common in females of from 11 months of age onwards. I have no figures now, but my recollection is that it developed in not less than 40 per cent of the older females. Also there was the odd occurrence in a male, though this was of course rare."

Further :

"This colony was allowed to die out in 1944, but in 1946 I obtained two females and a male from Dr. Bonser and started a new colony in the University of Sheffield. Since then a colony of some 200–300 mice has been maintained. . . . Thus I have had plenty of opportunity to observe the incidence of mammary carcinoma in older females, and I have found it to be common—much commoner it seems than in Dr. Bonser's parent strain in Leeds."

Further, in 1952 :

" The final chapter in the story starts in 1949 when, in the autumn, I took the milk factor out by fostering one family with a Strong's CBA female. The family was born in an open grid and the babies fell through into a lower compartment. My present colony is entirely derived from this one family, and now mammary cancer is exceedingly rare. Indeed I cannot remember when I last had a case. So my present stock of your mice is without the milk factor, and I suppose it is no use for your own present purposes."

I regret very much that I cannot definitely state so, but I have strong reasons to believe that the animals sent to Leeds in May, 1934, were descendants from the pair (4 + 5) of my " White Label ". This would offer a very natural explanation of the great similarity between the Leeds strain and one of the main branches of my strain under development in 1934. The animals sent to Leeds probably belonged to the $F_{16}$ of this branch, described above, and the mammary tumour characteristics are very similar. If this supposition is correct, it would establish a very formal and remote relationship only between the Oslo and the Leeds strains of " White Label ". Actually, they would have only the completely heterozygous founding mother in common.

Whereas the branch (4 + 5) has shown a remarkable stability from the very beginning until it became extinct in Oslo but was still maintained in England, the branch (2 + 3) at an early date started to segregate into very different lines with gradual stabilisation of their characteristics. The present only surviving line (426) in Oslo, through one influence or another, lost the milk factor probably at an early date. In a series of experiments a few years ago the milk factor was reintroduced into the Oslo " White Label " strain, through suckling a breast cancer female of another origin.

### SUMMARY.

A brief account is presented of the origin and the development of the " White Label " strain. Starting with a pregnant heterozygous female and through subsequent systematic brother-to-sister matings a non-selective segregation took place, resulting in the formation of two main branches and several lines. A description of some of these lines is given. At present one "White Label" strain (Leeds) carries the milk factor and produces about 30 per cent of mammary carcinomas in females reaching the age of 12 months or more. This strain has the characteristics of the branch (4 + 5) of the original material. Another " White Label " strain(Oslo) is devoid of the milk factor and produces practically no mammary carcinomas spontaneously. This strain is probably one of the lowest mammary cancer strains in existence. For future references it is therefore of great importance to distinguish between these two " White Label " strains ; therefore the Leeds strain will be designated WLL and the Oslo strain WLO. The two strains most probably have only a heterozygous founding mother in common.

This experiment has been carried out for more than twenty years. It would not have been possible without the assistance of my collaborator Miss Hj. Aasland, who continued the experiment during the first years of the war, until illness and an untimely death ended her devoted and loyal partnership. During the remaining years of the war the strain was maintained at a reduced scale through the initiative and faithful service of Mrs. E. Wraal.

The present paper has been prepared with financial aid from "Fylkesmann H. B. Guldahl og hustru Lucy Guldahls legat til bekjempelse av kreft og andre alvorlige sykdommer".

### REFERENCES.

KREYBERG, L.—(1935) *Amer. J. Cancer*, **24**, 554.—(1936) *Nord. med. Tidsskr.*, **12**, 1236.
STRONG, L. C.—(1942) *Cancer Res.*, **2**, 531.